# Developing a broad perspective of future work and career in medical students through field trips to a disaster area: a qualitative study

**DOI:** 10.1186/s13104-024-06724-9

**Published:** 2024-03-04

**Authors:** Tomoo Hidaka, Shota Endo, Hideaki Kasuga, Yusuke Masuishi, Takeyasu Kakamu, Tetsuhito Fukushima

**Affiliations:** Department of Hygiene and Preventive Medicine, Fukushima Medical University, Hikarigaoka 1, 960-1295 Fukushima City, Fukushima Japan

**Keywords:** Qualitative research, Group interview, Undergraduate medical education, Fukushima nuclear accidents, Social medicine, Human development, Career choice

## Abstract

**Objective:**

Field trips to disaster-affected areas (FTDAs) without a specific purpose, such as medical cooperation, are widely used in medical education. However, what medical students gain from FTDAs remains unclear. The present study aimed to clarify what medical students gain from FTDAs. Five medical students who had visited the Fukushima nuclear power plant in Japan participated in a semi-structured group interview to ask what they gained from such a visit. The narratives were analysed using open coding.

**Results:**

The following four themes emerged: “Spirit of scientific inquiry”, “Foundation for lifelong education and personal growth”, “Broadened understanding of the medical profession”, and “Importance of practicing medicine in the community setting”. The ambiguity of medical students’ specific roles in the field trip compared to the fieldwork may have encouraged them to make sense of the experience from their perspective. As a result, students may have gained a broader perspective of their future work and career through the FTDA. If medical educators can gain consensus from the residents of a disaster site, different disaster-affected areas can be potential sites for medical education using FTDAs.

## Introduction

Past studies suggested that visiting disaster-affected areas to interact with residents and see the effects of a disaster contributes to the growth of medical students such as enhanced psychological resilience and improved understanding of professional responsibility [1–2]. The learning method in which students visit the site without being assigned specific roles, such as providing medical care, could be referred to as a ‘field trip’ (FT), whereas the term ‘fieldwork’ could be an educational method where students may be assigned tasks such as medical collaboration or support.

Previous studies have suggested the medical educational benefits of FT in general, such as a better understanding of the concept of social determinants of health [3], and those of fieldwork, such as improved professionalism and social responsibility [1, 4]. In line with these educational benefits of FT and fieldwork, participation in field trips to disaster-affected areas (FTDAs) may provide valuable insights to help medical students improve their learning motivation and career options. However, little is known about what the medical student gains from FTDAs.

The Fukushima Daiichi Nuclear Power Plant (FDNPP), the site of an accident caused by the 2011 Great East Japan Earthquake (GEJE) and subsequent tsunami, and its circumstances are considered to be representative of disaster-affected areas in the post-disaster recovery period due to the severity of the damage caused to society. Therefore, the FT to the FDNPP is classified as an FTDA and assumed to be a suitable place to investigate what the medical student gains from FTDAs.

The present study aimed to investigate what the medical student gained from participating in FT to FDNPP using a group interview. Even though this is an exploratory study in a single area, essential implications can be drawn.

## Methods

### Recruitment

Using purposive sampling, the participants were five medical students from Fukushima Medical University (FMU) attending a six-week intensive course in social medicine, including the one-day FT to FDNPP. All participants were male, in their fourth year at the time of the FTDA, which is the last year before starting bedside learning at the hospital, and over 20 years of age, which is the legal age of majority in Japan. Participants included those from Fukushima Prefecture. However, none had suffered direct damage from the GEJE, such as injury to themselves or their family members or property loss due to the disaster.

In fiscal 2016, when the FTDA was conducted, there were 758 medical students at FMU. Of these, 147 were fourth-year students, five of whom participated in the FTDA.

### Research design and setting

The students participated in an FT to the FDNPP on 7 February 2017 and then in a retrospective group interview on 17 December 2018. The participants were, therefore, in their fourth year at the time of the FT and in their sixth year at the time of the interview.

The restoration and tours of the FDNPP were managed by the Tokyo Electric Power Company (TEPCO), which operates the FDNPP and has been accepting small groups of visitors since 2011, the year of the accident (2017 telephone interview with a TEPCO spokesperson by the first author after the FT to the FDNPP; unreferenced). TEPCO initially did not allow young people to visit the plant for health and safety reasons but relaxed the policy when radiation levels at the FDNPP site fell to non-hazardous levels in 2016. Safety was also assured in 2017 when the current study joined the FT.

The FT began with a briefing by a TEPCO spokesperson on the history of the accident and the recovery status. The visitors, including five students and four teachers, took a bus tour of the FDNPP premises. The route included areas with clear traces of the accident, such as the Unit 1 and Unit 2 buildings where the hydrogen explosions occurred and the coastline directly affected by the tsunami. After the bus tour, the visitors’ exposure dose was checked for safety reasons. Then, a question and answer session with TEPCO spokespersons was held. The whole FT lasted about four hours.

The subjects may have been mentally affected by the destruction of the FDNPP and the surrounding area during the FT. Therefore, a medical doctor monitored the students’ mental health during the FT in 2017 and confirmed the same in 2018 when the interviews were conducted. No problems were reported.

The retrospective group interview was not part of a regular curriculum; it was conducted in a context unrelated to formal educational assessment in the authors’ institution. Thus, the student’s grades or standing at the university would remain unaffected by their shared experiences.

### Interview procedure

A group interview was used to facilitate the subjects’ recall of past experiences. The interview was conducted in a conference room at FMU on 17 December 2018, with the first author as the moderator. The first author’s credentials and related information are listed in Table 1. The conversations were recorded with an IC recorder, with the participants’ permission, and a verbatim transcript was made. The group interview began with the question: “What have you gained from the FTDA experience? The interview lasted about 60 minutes.

**Table 1:** Credentials and related information of the authors

**Credentials of the first author**
**Gender**: Male
**Position**: Associate Professor in the Department and one of the teachers responsible for the six-week intensive course in social medicine, including FTDA.
**Experience and academic career**: The first author had a 15-year career as a researcher in the humanities and social sciences, specialising in qualitative research. He had experience in both participant observation and interview studies, and had published nine original research articles and one book. He had also been a lecturer on the social medicine fieldwork course for six years.
**Relationships between the authors and participants**
**Prior to the FTDA**: Of the five participants, three individuals had a previous relationship with the first author, as the first author was a lecturer in the social medicine fieldwork class prior to the FTDA in 2015. The first author got to know them during this class. The other two individuals met the authors for the first time during the six-week intensive course in social medicine.
**During the FTDA**: After completing the course assignments, as part of the pre-learning for the FTDA, the participants extensively studied papers and media reports on the FDNPP accident and occupational health issues for decontamination and reconstruction workers. All authors, including the first author, took part in this pre-learning as lecturers, which allowed students and authors to become familiar with each other.
**After the FTDA**: Even after the completion of the six-week intensive course in social medicine, which included the FTDA, participants voluntarily visited and stayed in the department when they had questions related to social medicine, occupational health or medical statistics. Through these interactions, a continuous relationship was established from the time of the intensive course to the retrospective group interviews for the present study.

### Analytic procedure

The transcript was inductively coded according to open coding, whereby labels were assigned to fragments of the transcript that had similar underlying meanings, and axial coding, whereby the meanings and interrelationships of labels/categories were examined [5]. The student’s reports and conversations were summarised in three steps. Starting with 67 narrative segments, the number of labels in each step was 15, 8 and 4. The concepts in step four were treated as the ‘main theme’, which reflected what the subjects had gained from the FT, and eight corresponding ‘sub-themes’ in step three, which provided more concrete explanations of the subjects’ evaluations of their experiences of the FT than the main themes.

The coding procedure was as follows. First, the first author conducted open and axial coding, producing an analytical draft with a code tree; the code tree organised the hierarchical relationships between labels and categories. Second, the third and fifth authors confirmed this draft for appropriateness of procedure, content and wording by asking the first author about the reasons and criteria for coding; the authors carefully checked whether the labels sufficiently reflected the medical students’ insights and examined the coding for arbitrariness. Thirdly, the third author, an experienced qualitative researcher, checked the adequacy of the analysis, and the fifth author contributed to the elaboration of the content and interpretation of the main and sub-themes through his profession as a medical doctor who knew the history and trends of medical education.

## Results

The main and sub-themes and excerpts of narratives are shown in Table 2.

**Table 2:** Themes and their detailed explanations about what medical students gained from the FT

Main theme	Sub-theme	Definition	Narrative example
Spirit of scientific inquiry	Ability to perform objective judgment toward radiation health risk	The ability to judge truth or falsity toward the topic of radiation health risk and radiation management, critically examining current information such as media reports.	“To be honest, I was afraid to visit the FDNPP since I heard negative topics of the FDNPP from media. When I actually visited the site and checked the dose rates during the tour, I found that, from a scientific point of view, the dose rates were not high enough to cause harm to the human body.” (Subject B) “I was interested in the radiation management of the FDNPP restoration workers. I assume that each worker has a different background, such as level of education, so how have they been trained? The appropriate training may be different depending on the level of education. Without such appropriate training, the health of the workers may be at high risk. Beyond the explanation of the spokespersons, we need objective information to judge the risk of radiation and quality radiation management. This is what I knew through the FTDA.” (Subject D)
	Field-based knowledge of FDNPP restoration situation	Updated knowledge of the status of FDNPP restoration based on actual field observation (FTDA), taking into account the consequences of the restoration.	“Visiting a nuclear power plant was a special experience for me. My local friends were losing interest in the FDNPP accident and its recovery, so I was able to revive their interest by telling them about my experience of visiting the plant, such as the reality of radiation protection, the fact that the radiation dose was lower than expected, and the expectation that future recovery will not be an easy process. It was a good experience for me to know what the situation was really like at the FDNPP.” (Subject A) “During the FT to the FDNPP, I was introduced to groundwater and sewage water control equipment to prevent radioactively contaminated water from leaking into the ocean, such as ALPS*, waterproof walls, and groundwater bypasses. I was surprised that such technologies actually existed and were being used. However, there is still a lot of contaminated water and materials temporarily stored on the premises of the FDNPP that have not yet been disposed of. I thought their disposal would be a significant issue in the future.” (Subject B) *Note: ALPS (Advanced Liquid Processing System) is a system to remove radioactive materials from contaminated water, reducing the radiation dose to meet national safety standards by chemical and physical technologies.
Foundation for lifelong education and personal growth	Deeper understanding of the appeal of public health and preventive medicine	Discovery and/or enhanced understanding of the appeal of occupational health and preventive medicine from the viewpoint of practice.	“I have taken many clinical courses in my practical training, for example, the bedside learning program. However, it was not until I took part in the FT that I fully understood preventive medicine or public health. I gained an understanding that taking care of workers’ safety, for example, preventing industrial accidents is one of the central issues in preventive medicine and occupational/public health. In order to support the health of workers, we need to know about their backgrounds, such as their working conditions and education level. It may become my life’s work.” (Subject B) “The work of doctors is often thought of as ‘curing disease’, or so I thought. That may be true. However, occupational health and preventive medicine are about helping people to be more fulfilled in their daily lives and work. It was good to know there are ways to provide this kind of health care and its attractiveness.” (Subject C)
	Enjoyment of problem finding and solving	Recognition of the fun in problem finding and solving itself, not just in the context of medical practice.	“I have been interested in public health since my fourth year at university. I enjoy and like thinking about health problems and how to tackle them using statistics. The FT has allowed me to talk to people, not just statistics. When you come into contact with data, whether numbers or stories, you get to know new aspects of people. It’s an exciting process to think about what the problem is and how I can improve it.” (Subject C) “I learned the advantages and fun of FTs and interviews. These methods are quite attractive for finding health issues and solutions for workers. It was my first time joining a practical training like the FT to the FDNPP, but I enjoyed it. This is a unique educational course.” (Subject D)
Broadened understanding of the medical profession	Career path to an ideal doctor	A broadened view of how to work as a medical doctor, with the conviction that a medical doctor can improve health problems by understanding the social background and life of their patient.	“My experience of the trip to the FDNPP had a lasting impression on me. There is a way of being a doctor who may not directly cure patients but can contribute to workers’ health by paying close attention to the background. I think such a background is called humanity: the way of living and human relationships established by the workers. Such attention to the background is essential for an eventual understanding of the person.” (Subject A) “When I see patients in surgical training, I don’t really look at the patient’s background. The FT, on the other hand, allowed me to focus on the patient’s humanity. It was certainly very different from learning in the classroom. As a surgeon, and in other areas of medicine, you should be interested in the patient’s life. I want to keep that perspective.” (Subject C)
	Mental preparation by observation to clarify the ideal way to proceed	Reflections on how to work as a medical professional in the future, based on the working practices and health support needs of the people met through FT.	“Being a doctor and dealing with a patient who gets sick is indeed good. But I have been exposed to preventive medicine and its idea. If I can save thousands of people before they get sick, that is a better way. In the future, I will meet patients and residents who should be treated with the idea of prevention. This training (a six-week intensive course in social medicine) may have been a preparation for that. This is a very promising future path for me.” (Subject A) “In my case, before I entered medical school, I thought that the main job of a medical doctor was to perform surgery. However, I learned various things after entering school and saw different paths. Honestly, I have decided on the department I want to enter, and the department has nothing to do with disaster medicine or radiology. After I get into it, I may develop an interest in other things. This development should be taken as a positive sign. The FT to the FDNPP allowed me to discover such positive viewpoints and possible career options.” (Subject C)
	Variety of ways doctors can support society	Insights into how medical doctors contribute to society, focusing on the status and value they hold in society rather than on their competencies, such as knowledge and skills.	“It is rewarding to serve others, but that service is not limited to being thanked for curing illnesses. A medical doctor may contribute to an organisation with an installation criterion in law that requires the presence of a medical doctor just by belonging to it since the organisation must need a professional with a license. In this way, if I can be useful as a doctor by helping others, I can contribute in various ways.” (Subject B) “For better or worse, even if you say the same thing, it can be more convincing when a doctor says it. Doctors need to see that they have such a position in society. It is not just about curing diseases; by showing up as a doctor and communicating with patients, patients may be able to feel health advice as reassuring and convincing. Doctors take advantage of their strong position, but it makes sense if they can realistically contribute to health. There are many ways in which a doctor can be helpful to society.” (Subject D)
Importance of practicing medicine in the community setting	The reality of medically underserved areas as a potential workplace	A sense of one’s responsibility or mission considering the social situation regarding medical care in the disaster-affected area.	“There is a new hospital in the city near the FDNPP. This hospital is closely linked to the university’s medical department. If I become a professional doctor and work at the university, I could be sent to this hospital. Even within the same prefecture, this area has a smaller population and fewer medical resources than this area (where the university is located), so there are specific challenges. There are many older adults, and there is a need for occupational health care for the recovery workers. If I work at this hospital, I need to address these challenges.” (Subject B) “I had to go to the Hamadori area* in Fukushima Prefecture for practical training after this laboratory assignment program. From the practical training, I realised the severity of medically underserved areas, and I thought that the FDNPP greatly impacted the area. After graduation, I plan to work in Fukushima Prefecture, so I may stay and work in the Hamadori area. If I do, the impact of the FDNPP accident will be related to my work, although it may be indirect.” (Subject D) * Note: The Hamadori area is the coastal region of the Prefecture on the east side. It is the region where the FDNPP is located and is faced with limited medical resources.

Note: For the main theme, “Importance of practising medicine in the community setting,” there was only one corresponding sub-theme; we generated this sub-theme and then labelled it as a main theme with the equivalent level of abstraction as the other main themes. The students’ accounts were quoted to support the credibility of each theme; the students’ names were represented by the letters A to D to anonymise them

### Spirit of scientific inquiry

The main theme, “Spirit of scientific inquiry,” represents the knowledge and mindset required to critically read and examine the prevailing notions and government’s pronouncements about the destruction and reconstruction after the disaster, the FDNPP accident.

### Foundation for lifelong education and personal growth

The main theme, “Foundation for lifelong education and personal growth,” emphasises that the FTDA experience has been associated with a recognition of the importance of lifelong learning, an increased interest in public health and preventive medicine to reduce the risk of radiation exposure among FDNPP clean-up workers, and the enjoyment of problem-solving.

### Broadened understanding of the medical profession

The main theme, “Broadened understanding of the medical profession”, indicates that students have gained a broader insight into the essential elements of becoming a doctor and different perspectives on their future career choices than before joining the FT.

### Importance of practicing medicine in the community setting

In the main theme, “Importance of practising medicine in the community setting”, the students expressed their recognition of their future vision as medical practitioners. They mentioned the possibility of working in a region surrounding or close to the FDNPP and the feeling of having to contribute to the region.

## Discussion

### Findings

Our findings suggest that FT had a clarifying effect on students’ future perspectives, such as broadening their career options. The ambiguity of medical students’ specific roles in FT compared to fieldwork may have encouraged them to make sense of the experience from their perspective; in other words, the medical educational effects of fieldwork in disaster areas indicated by past studies, such as psychological growth [2, 6], professionalism and increased social responsibility [1, 4], may be determined by the presence of specific roles. Our findings on what the medical student gains from FTDA were novel and different from those of FT in general shown by previous studies, such as promoting understanding of the social determinants of health and refining professional knowledge [3, 7, 8].

### Methodological implication

Regarding the methodology of the present study, we should mention that the retrospective group interviews conducted two years after the FT, 2019 may have helped the medical students to recall their past experiences and make sense of their studies; in other words, there may have been a ‘booster effect’ or promotion of effectiveness in their medical education. Although the data collection method may have inevitably acted as an intervention, we believe that this method contributed to clarifying the students’ past experiences rather than compromising the quality of the study design. It is suggested that an FTDA has an enhanced educational effect when students reflect on it over time.

### Generalisability/transferability

We acknowledge that the generalisability of our findings is limited because the subjects of this study were selected from a single medical university. However, it should be noted that the concept of transferability is more appropriate for qualitative research than generalisability in quantitative research; transferability is defined as the extent to which findings from one setting can be found in another setting [9]. The main and sub-themes in the present study were generated from the narratives through a step-by-step coding analysis procedure; these themes were relatively abstract and may be common to FTDA cases in other settings. Therefore, we believe that our findings are transferable.

### Applicability

Areas affected by disasters such as floods, volcanic eruptions and landslides can become sites for FTDAs if local residents are willing. Importantly, FTDAs should not become disaster tourism, which is a selfish and opportunistic visit to a disaster-affected area that lacks consideration and respect for the local situation and residents [10, 11] and can be described as “medical shame“ [12]. FTDAs should be conducted after building trust with local residents to avoid disaster tourism. Universities should explain the meaning and purpose of FTDAs to residents of disaster areas in order to build a trusting relationship with the local community for sustainable medical education in a region.

## Limitations

The limitations of the present study are that there is room to explore how educational indicators such as career choices and learning outcomes are related to what students gain from the FTDA; such exploration may be a more practical contribution to medical education than the present study. Furthermore, in qualitative research using interview, it is generally desirable to explore the psychological state or transition of the participant in more depth by providing multiple interview opportunities; however, in the present study, data were obtained through a single group interview. A longitudinal study should be conducted to reveal the transition process of medical students’ growth. In terms of credibility, one of the criteria of a qualitative study is trustworthiness; the present study may need improvement in this regard. Future research should use the analytical option of triangulation to increase methodological rigour and trustworthiness. As an example of methodological triangulation, a psychological scale could be used to verify the consistency of interpreting the qualitative study results from a future perspective. However, this was not feasible in this study as a suitable scale could not be identified. Achieving data saturation and implementing participant check to analytical results will also be required in future studies.

## Data Availability

The datasets generated and/or analyzed during the current study are not publicly available due to the inclusion of personally identifiable information but are available from the corresponding author on reasonable request. The data will be provided in a de-identified manner.

## Abbreviations

FT: Field trip
FTDA(s): Field trip to disaster-affected area(s)
FMU: Fukushima Medical University
FDNPP: Fukushima Daiichi Nuclear Power Plant

## References

[1] Reyes H (2010). Students’ response to disaster: a lesson for health care professional schools. Ann Intern Med.

[2] Kaye-Kauderer HP, Levine J, Takeguchi Y, Machida M, Sekine H, Taku K, Yanagisawa R, Katz C (2019). Post-traumatic growth and resilience among medical students after the March 2011 disaster in Fukushima, Japan. Psychiatr Q.

[3] Chang AY, Bass TL, Duwell M, Berger JS, Bangalore R, Lee NS, Amdur RL, Andrews M, Fahnestock E, Kahsay L, El-Bayoumi J (2017). The impact of see the City you serve field trip: an educational tool for teaching social determinants of health. J Grad Med Educ.

[4] Bazan D, Nowicki M, Rzymski P (2021). Medical students as the volunteer workforce during the COVID-19 pandemic: Polish experience. Int J Disaster Risk Reduct.

[5] Holton JA, Bryant A, Charmaz K (2007). The coding process and its challenges. The sage handbook of grounded theory.

[6] Anderson D, Prioleau P, Taku K, Naruse Y, Sekine H, Maeda M, Yabe H, Katz C, Yanagisawa R (2016). Post-traumatic stress and growth among medical student volunteers after the March 2011 disaster in Fukushima, Japan: implications for student involvement with future disasters. Psychiatr Q.

[7] Friedland AR, Rintel-Queller HC, Unnikrishnan D, Paul DA (2012). Field trips as a novel means of experiential learning in ambulatory pediatrics. J Grad Med Educ.

[8] Hartman M, Thomas S, Ayoob A (2018). Radiology field trips––a list of must sees in the radiology department for medical students: how we do it. Acad Radiol.

[9] Wang S, Moss JR, Hiller JE (2006). Applicability and transferability of interventions in evidence-based public health. Health Promot Int.

[10] Civaner MM, Vatansever K, Pala K (2017). Ethical problems in an era where disasters have become a part of daily life: a qualitative study of healthcare workers in Turkey. PLoS ONE.

[11] Rucinska D (2016). Natural disaster tourism as a type of dark tourism. Int J Hum Soc.

[12] Van Hoving DJ, Wallis LA, Docrat F, De Vries S (2010). Haiti disaster tourism: a medical shame. Prehosp Disaster Med.

